# Phosphatidylserine exposure modulates adhesion GPCR BAI1 (ADGRB1) signaling activity

**DOI:** 10.1016/j.jbc.2022.102685

**Published:** 2022-11-09

**Authors:** Trisha Lala, Juleva K. Doan, Hiroyuki Takatsu, H. Criss Hartzell, Hye-Won Shin, Randy A. Hall

**Affiliations:** 1 Department of Pharmacology and Chemical Biology, Emory University School of Medicine, Atlanta, Georgia, USA; 2 Department of Physiological Chemistry, Graduate School of Pharmaceutical Sciences, Kyoto University, Sakyo-ku, Kyoto, Japan; 3 Department of Cell Biology, Emory University School of Medicine, Atlanta, Georgia, USA

**Keywords:** brain, G protein–coupled receptor, signal transduction, lipid-binding protein, cell culture, synaptic, engulfment, dimerization, multimerization, externalization, AGPCR, adhesion G protein–coupled receptor, ANO6, anoctamin 6, B1FL, full-length B1, co-IP, coimmunoprecipitation, CNS, central nervous system, CTF, C-terminal fragment, EV, empty vector, GAIN, GPCR autoproteolysis–inducing domain, GPCR, G protein–coupled receptor, HA, hemagglutinin, HEK293T, human embryonic kidney 293T cell line, NTF, N-terminal fragment, PEI, polyethyleneimine, PS, phosphatidylserine, PSD, postsynaptic density, TM, transmembrane, TSR, thrombospondin repeat

## Abstract

Brain-specific angiogenesis inhibitor 1 (BAI1; also called ADGRB1 or B1) is an adhesion G protein–coupled receptor known from studies on macrophages to bind to phosphatidylserine (PS) on apoptotic cells *via* its N-terminal thrombospondin repeats. A separate body of work has shown that B1 regulates postsynaptic function and dendritic spine morphology *via* signaling pathways involving Rac and Rho. However, it is unknown if PS binding by B1 has any effect on the receptor’s signaling activity. To shed light on this subject, we studied G protein–dependent signaling by B1 in the absence and presence of coexpression with the PS flippase ATP11A in human embryonic kidney 293T cells. ATP11A expression reduced the amount of PS exposed extracellularly and also strikingly reduced the signaling activity of coexpressed full-length B1 but not a truncated version of the receptor lacking the thrombospondin repeats. Further experiments with an inactive mutant of ATP11A showed that the PS flippase function of ATP11A was required for modulation of B1 signaling. In coimmunoprecipitation experiments, we made the surprising finding that ATP11A not only modulates B1 signaling but also forms complexes with B1. Parallel studies in which PS in the outer leaflet was reduced by an independent method, deletion of the gene encoding the endogenous lipid scramblase anoctamin 6 (ANO6), revealed that this manipulation also markedly reduced B1 signaling. These findings demonstrate that B1 signaling is modulated by PS exposure and suggest a model in which B1 serves as a PS sensor at synapses and in other cellular contexts.

G protein–coupled receptors (GPCRs) are a diverse superfamily of receptors characterized by a conserved seven-transmembrane (TM)-domain architecture. Given that over 500 Food and Drug Administration–approved drugs and almost 100 drug candidates in clinical trials target GPCRs, there is great interest in the elucidation of the pharmacology of orphan GPCRs that lack well-defined ligands (1). Adhesion GPCRs (AGPCRs) are one of five major GPCR families, and most receptors in this family are still considered to be orphans (2, 3, 4). Members of the AGPCR family play crucial roles in a myriad of physiological processes, and several clinical disorders are associated with the dysfunction of this receptor type (2, 3, 4). Thus, pharmacological modulation of these receptors has the potential to provide powerful new therapeutics.

AGPCRs derive their name from the adhesive properties of the receptor class, which are conferred by their large extracellular N-terminal fragments (NTFs). Most members of this receptor family undergo autoproteolytic cleavage *via* a conserved GPCR autoproteolysis-inducing (GAIN) domain, which cleaves the NTF from the C-terminal fragment (CTF) that contains the seven-TM region (5). Following GAIN-mediated cleavage, the resultant NTF and CTF remain noncovalently associated for some period, with this interaction inhibiting downstream signaling by the CTF. The engagement of the NTF by extracellular ligands may either remove the NTF from the CTF or cause conformational changes in the CTF that activate downstream signaling (2, 3, 4).

The AGPCR known as brain-specific angiogenesis inhibitor 1 (BAI1; also known as ADGRB1 or B1) was originally discovered as a thrombospondin repeat (TSR)–containing receptor enriched in the brain and capable of modulating angiogenesis when overexpressed (6). While B1 remains an orphan receptor, a seminal article by Ravichandran *et al.* (7) revealed that the TSRs of B1 can bind to phosphatidylserine (PS) to facilitate the engulfment of apoptotic cells by macrophages. Subsequently, other physiological roles for B1 in macrophages have also been elucidated, such as the binding of Gram-negative bacteria to facilitate their engulfment (8, 9, 10, 11).

Parallel to this work in macrophages, there exists a completely separate literature of studies by multiple groups on B1 regulation of brain physiology. B1 is enriched in the postsynaptic density (PSD) and regulates the morphology of dendritic spines in cultured neurons (12, 13, 14, 15). Mice lacking B1 exhibit reduced PSD thickness, disrupted synaptic plasticity, impaired spatial learning, and social deficits (16, 17). B1 has been shown to stimulate RhoA signaling *via* coupling to both Gα12/13 (13, 18) and Bcr (15) and to in addition promote Rac1 signaling *via* coupling to Tiam1 (12, 14) in transfected cells and cultured neurons. However, it is unknown whether B1 activation of any of these signaling pathways is influenced by B1 binding to PS.

We sought to connect the work done on PS binding by B1 in macrophages with the literature on B1 signaling in the nervous system. PS is normally found in the inner leaflet of the plasma membrane, and the asymmetric distribution of PS is maintained by a class of enzymes known as flippases, which are P4 ATPases that actively translocate PS from the outer leaflet of the plasma membrane to the inner leaflet (19). Cellular stress can promote PS externalization to the outer leaflet of the plasma membrane by inhibiting flippases and/or activating transporters known as floppases and scramblases that move lipids in the opposite direction to the flippases (19). Externalization of PS is known to occur during apoptosis, but it is also now well appreciated that PS externalization can occur under normal physiological conditions and serve as an important cellular signal in the nervous system (and other systems) that leads to pleiotropic effects depending on how the signal is decoded by various PS-binding receptors (20, 21, 22).

We leveraged recent advances in the understanding of PS biology to create cellular conditions in which B1 would encounter differing levels of PS exposure in the outer leaflet. We hypothesized that B1 interaction with externalized PS, embedded in the plasma membrane, might induce conformational changes in the B1 NTF and thereby modulate B1 signaling.

## Results

### Human embryonic kidney 293T cells exhibit a baseline level of exposed PS that can be modulated by the PS flippase ATP11A

To assess whether exposure of PS can modulate B1 signaling, we developed a cell culture model in which levels of exposed PS could be reproducibly manipulated. B1 signaling has previously been studied in human embryonic kidney 293T (HEK293T) cells (13, 18), which are known to express an endogenous scramblase, anoctamin 6 (ANO6/TMEM16F) (23). The native presence of this scramblase results in a measurable population of HEK293T cells in culture being PS+ (meaning that a quantifiable amount of PS in the outer leaflet of the plasma membrane can be measured *via* annexin V binding) under most growth conditions (23). HEK293T cells also express endogenous CDC50A (24, 25), a chaperone protein that is required for the function of ATP11 family of flippases (26). Thus, given that HEK293T cells exhibit a basal level of PS exposure and also express the machinery needed for PS flippase function, they represented an attractive model for our studies.

**Figure 1:**
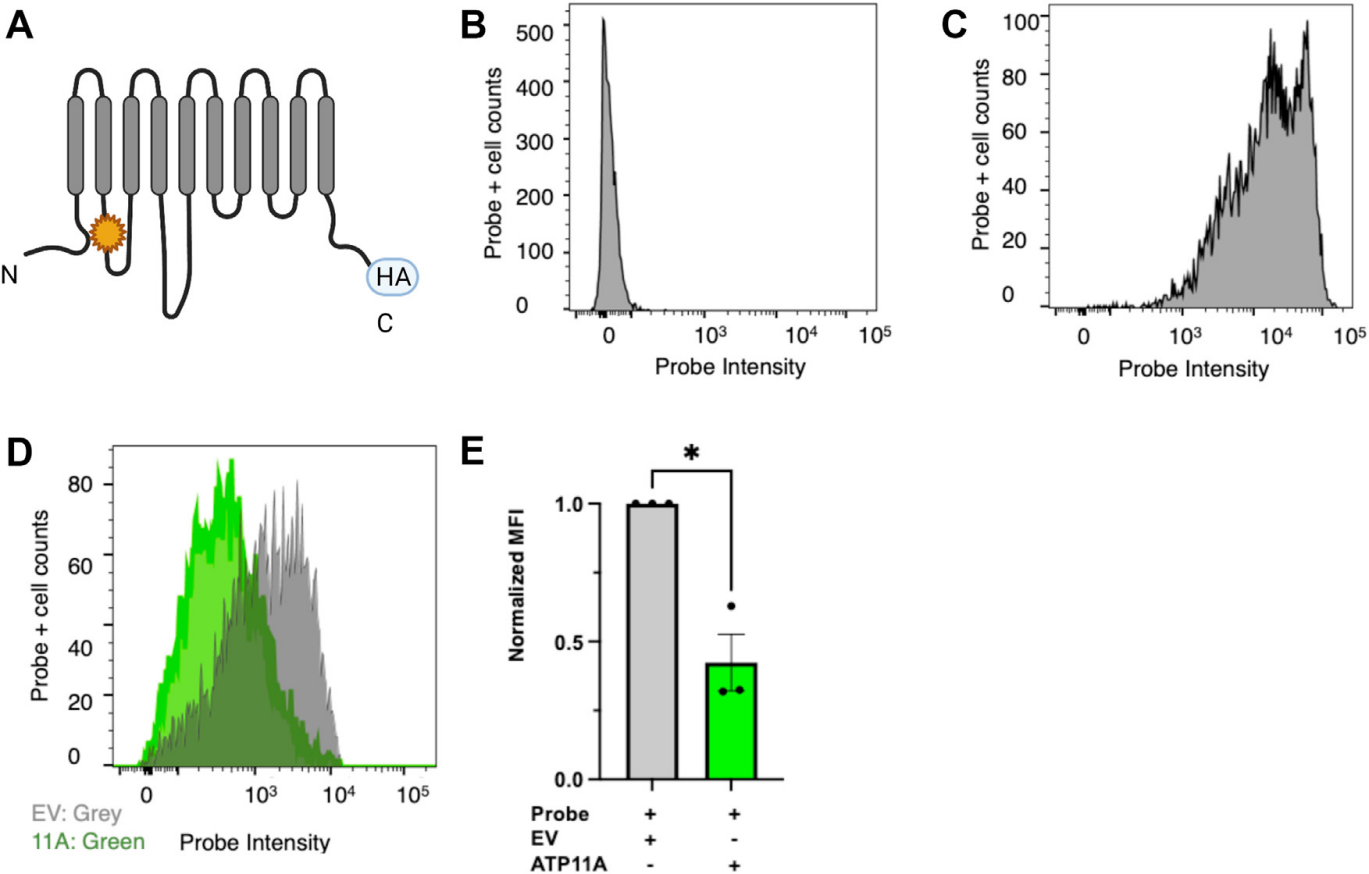
**Evaluation of phosphatidylserine (PS) exposure in HEK293T cells at baseline and when overexpressing ATP11A**. Flow histograms of annexin V binding (in relative units) *versus* cell counts are shown; 20,000 cells were counted. *A*, ATP11A schematic: depiction of the PS flippase, ATP11A. *Star* indicates wildtype flippase. HA tag location is shown. *B*, negative control: flow histogram of mock-transfected HEK293T cells (−annexin V) demonstrates low nonspecific signal. *C*, positive control: flow histogram of mock-transfected HEK293T cells (+annexin V) treated with 10 μM A23187 for 20 min to induce scramblase activity, resulting in high levels of PS exposed. *D*, ATP11A-induced reduction in PS: flow histogram of mock-transfected (*gray*) HEK293T cells (+annexin V) overlaid with ATP11A-transfected (*green*) HEK293T cells (+annexin V) demonstrates that these cells exhibit baseline exposure of PS that can be reduced *via* overexpression of ATP11A. *E*, Quantification of ATP11A-induced reduction in PS exposure: mean fluorescence intensity (MFI) shown with ATP11A+ condition normalized to mock-transfected condition. ATP11A overexpression in HEK293T cells resulted in a 58% reduction in PS exposure (mean ± SEM shown, unpaired *t* test, *p* = 0.03, n = 3). HA, hemagglutinin; HEK293T, human embryonic kidney 293T cell line.

We investigated PS exposure in HEK293T cells using flow cytometry to assess whether overexpression of ATP11A, a phospholipid flippase, could modulate the levels of PS exposed on the outer leaflet in these cells. Figure 1*A* is a schematic diagram of ATP11A, which is a large 10-TM protein with both its N- and C-terminal regions in the cytoplasm. To visualize externalized PS, the cells were incubated with an annexin V probe; in the absence of the probe, no measurable signal could be detected (Fig. 1*B*). In contrast, when HEK293T cells were treated with 10 μM of the calcium ionophore A23187 to strongly activate endogenous scramblases such as ANO6, we found that the annexin V probe detected very high levels of exposed PS in the A23187-treated HEK293T cells (Fig. 1*C*). We next measured baseline levels of PS exposure in HEK293T cells. As expected based on previous reports (23), the HEK293T cells at baseline exhibited a measurable level of PS exposure. Moreover, we found that the levels of externalized PS could be reduced *via* overexpression of ATP11A. Figure 1*D* compares baseline PS exposure in wildtype cells *versus* ATP11A-transfected cells. These studies revealed that HEK293T cells exhibit a quantifiable amount of externalized PS at baseline, and that overexpression of the PS flippase ATP11A in this cell type dramatically reduces PS exposure (total reduction = 58%; quantification is shown in Fig. 1*E*).

### Coexpression of ATP11A with B1 reduces the constitutive signaling activity of B1

**Figure 2:**
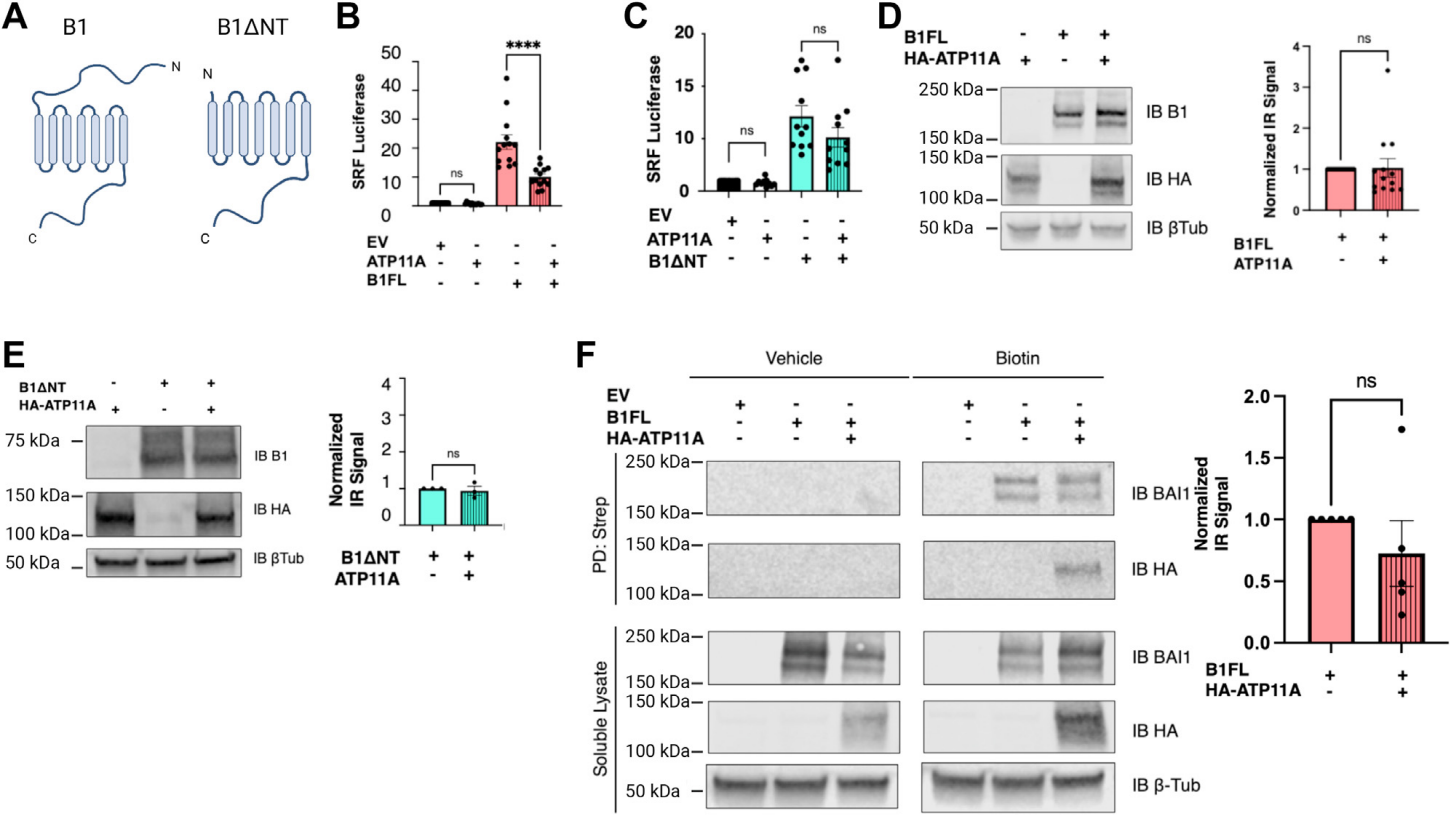
**ATP11A coexpression reduces B1 signaling activity.***A*, B1 schematics: depiction of B1FL on *left*, shown with full NTF, and B1ΔNT on *right*, lacking NTF up to site of predicted GAIN domain cleavage. *B*, coexpression with B1FL in HEK293T cells resulted in 54% reduction in B1FL activation of SRF-luciferase (mean ± SEM shown, ordinary one-way ANOVA with Tukey’s multiple comparisons test, *p* < 0.0001, n = 13, ROUT method used at 10% to remove two outliers). “IB" refers to “immunoblot” to indicate what antibody was used to detect the protein bands shown *via* Western blot. *C*, ATP11A coexpression with B1ΔNT resulted in no significant change in receptor activation of SRF-luciferase (mean ± SEM is shown, ordinary one-way ANOVA with Tukey’s multiple comparisons test, n = 11, ROUT method used at 10% to remove one outlier). *D*, ATP11A coexpression with B1FL did not significantly alter total cell lysate expression levels of receptor. Representative Western blot shown on *left* with quantification on *right* (normalized mean ± SEM is shown, unpaired *t* test, n = 13). *E*, ATP11A coexpression with B1ΔNT did not significantly alter total cell lysate expression levels of receptor. Representative Western blot shown on *left* with quantification on *right* (normalized mean ± SEM is shown, unpaired *t* test, n = 3). *F*, ATP11A coexpression with B1FL did not significantly alter receptor surface expression. Representative Western blot is shown on *left* with quantification on *right* (normalized mean ± SEM is shown, unpaired *t* test, n = 5). B1FL, full-length B1; GAIN, G protein–coupled receptor autoproteolysis–inducing domain; HEK293T, human embryonic kidney 293T cell line; NTF, N-terminal fragment.

We next investigated whether coexpression of ATP11A with B1 might modulate the G protein–dependent signaling of the receptor. It has previously been shown that full-length B1 (B1FL) expressed in HEK293T cells exhibits high constitutive signaling activity (13, 18). It is plausible that at least some portion of this activity may be dependent on stimulation of B1 signaling by baseline levels of PS exposure. Thus, we performed SRF-luciferase assays to assess B1FL coupling to Gα12/13 in control cells and cells coexpressing ATP11A. To investigate whether B1 signaling activity might be sensitive to PS exposure in a TSR-dependent manner, we also tested B1ΔNT, which lacks the NTF of the receptor and therefore lacks the TSRs that bind to PS (Fig. 2*A*).

Coexpression of B1FL with ATP11A reduced G protein–dependent signaling activity by 54% (Fig. 2*B*, one-way ANOVA with Tukey’s multiple comparisons test, *p* < 0.0001). In comparison, the G protein–dependent signaling of B1ΔNT was not significantly altered by coexpression with ATP11A (Fig. 2*C*), suggesting that the NTF of B1 is required for the impact of ATP11A on the signaling activity of the receptor. To determine whether the effect of ATP11A on B1 stimulation of SRF-luciferase was due to an overall reduction in the expression of the receptor, we measured B1 expression in the absence and presence of ATP11A and found that ATP11A does not alter the total protein levels of either B1FL (Fig. 2*D*) or B1ΔNT (Fig. 2*E*). We also assessed B1 surface expression and found that ATP11A had no significant effect on trafficking of B1FL to the plasma membrane (Fig. 2*F*). These findings demonstrated that the presence of ATP11A reduces B1 signaling activity but not the total or surface expression of the receptor.

### The flippase activity of ATP11A is required for modulation of B1 signaling

**Figure 3:**
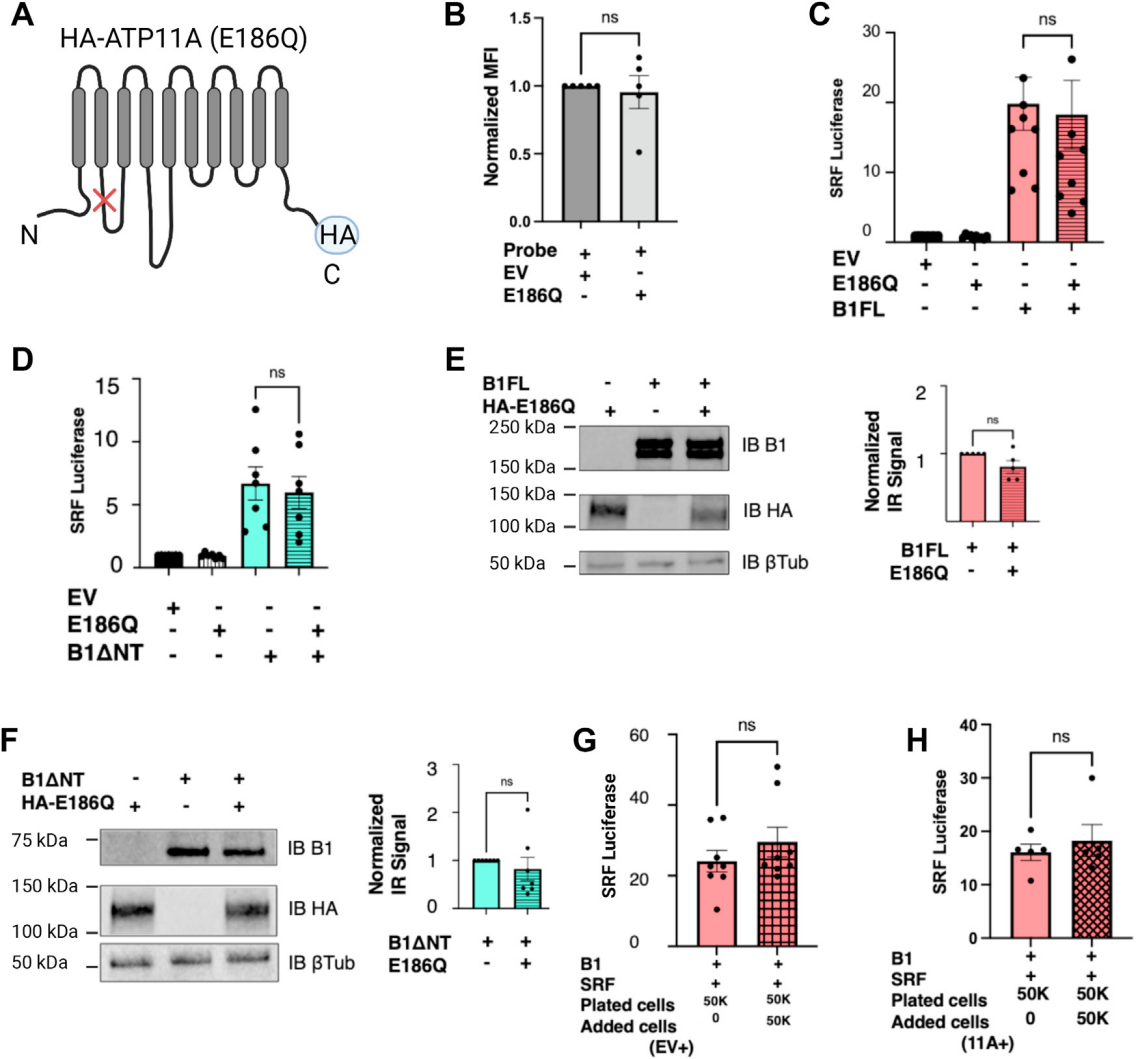
**Flippase-null mutant ATP11A (E186Q) does not alter B1 signaling activity.***A*, ATP11A-E186Q schematic*:* Depiction of mutant ATP11A at position 186 from E to Q that abolishes the flippase function. “X” shows location of the mutation, and the position of the HA tag is also shown. *B*, quantification of PS exposure in HEK293T cells overexpressing E186Q mutant: mean fluorescence intensity (MFI) shown with E186Q+ condition normalized to mock-transfected condition. E186Q overexpression in HEK293T cells resulted in no significant reduction in PS exposure (mean ± SEM is shown, unpaired *t* test, n = 5). *C*, ATP11A-E186Q coexpression with B1FL in HEK293T cells resulted in no change in B1FL activation of SRF-luciferase (mean ± SEM is shown, ordinary one-way ANOVA with Tukey’s multiple comparisons test, n = 10). *D*, ATP11A-E186Q coexpression with B1ΔNT also resulted in no significant change in receptor activation of SRF-luciferase (mean ± SEM is shown, ordinary one-way ANOVA with Tukey’s multiple comparisons test, n = 7). *E*, ATP11A-E186Q coexpression with B1FL did not significantly alter total cell lysate expression levels of receptor. Representative Western blot shown on *left* with quantification on *right* (normalized mean ± SEM is shown, unpaired *t* test, n = 5). *F*, ATP11A-E186Q coexpression with B1ΔNT also did not significantly alter total cell lysate expression levels of receptor. Representative Western blot shown on *left* with quantification on *right* (normalized mean ± SEM is shown, unpaired *t* test, n = 7). *G*, in comparison to baseline signaling of B1FL (*pink bar on left*), addition of 50,000 additional mock-transfected HEK293T cells did not significantly alter B1FL activation of SRF-luciferase (*pink and checkered bar on right*; mean ± SEM is shown, unpaired *t* test, n = 8). *H*, similarly, in comparison to baseline signaling of B1FL (*pink bar on left*), addition of 50,000 additional ATP11A-transfected HEK293T cells did not significantly alter B1FL activation of SRF-luciferase (*pink and crisscrossed bar on right*; mean ± SEM is shown, unpaired *t* test, n = 5). B1FL, full-length B1; HEK293T, human embryonic kidney 293T cell line; HA, hemagglutinin; PS, phosphatidylserine.

To dissect the mechanism of ATP11A-mediated regulation of B1 signaling activity, we next investigated whether the flippase function of ATP11A was required for the impact on B1FL signaling. For these studies, we utilized a mutant version of ATP11A that has a glutamate residue changed to glutamine at position 186 (E186Q). This mutation abolishes ATP11A flippase activity but does not affect the protein’s localization in the plasma membrane (26, 27, 28, 29). Figure 3*A* depicts the position of the ATP11A-E186Q mutation with an “X.”

Using flow cytometry, we first confirmed that this flippase-null mutant was indeed unable to significantly alter PS levels in HEK293T cells (Fig. 3*B*). Next, we performed SRF-luciferase signaling assays like those described previously, but in this case observed no significant impact of ATP11A-E186Q coexpression on the signaling activity of either B1FL or B1ΔNT (Fig. 3, *C* and *D*). The E186Q mutant also did not have any impact on the total expression of B1 (Fig. 3, *E* and *F*). These findings showed that the PS flippase function of ATP11A is required for modulation of B1 signaling activity.

### Increased cell density does not promote B1 signaling

The dependence on the flippase function of ATP11A for the protein’s effect on B1FL suggested that B1 detection of PS was stimulating receptor signaling. However, it was unclear whether the B1/PS interactions promoting signaling occurred on the same cell (*cis*) or between cells (*trans*). This question is important in understanding how PS might modulate B1 signaling physiologically. If B1 was predominantly detecting PS in *trans* on neighboring cells, then increasing cell density should magnify the effect and promote B1 signaling. Thus, we developed a coculture assay in which the number of B1/SRF-luciferase–transfected cells was held constant, but additional untransfected HEK293T cells were added to some of the wells to determine whether cell density could modulate B1 signaling. As shown in Figure 3*G*, we observed that B1FL signaling activity was largely unaffected by coculturing the B1/SRF-luciferase–transfected cells with additional untransfected cells. We repeated the same coculture assay, but this time we added ATP11A-transfected HEK293T cells. This modified coculture condition also did not modulate B1 signaling, providing evidence that B1 and ATP11A need to be expressed in the same cells in order for ATP11A to modulate B1 signaling (Fig. 3*H*). These observations together suggest that B1FL does not predominantly detect PS in *trans* in this system but rather likely detects PS in the same cell, at least under the conditions of these experiments.

### B1 multimerizes *via* its TM domains in a PS-independent manner

**Figure 4:**
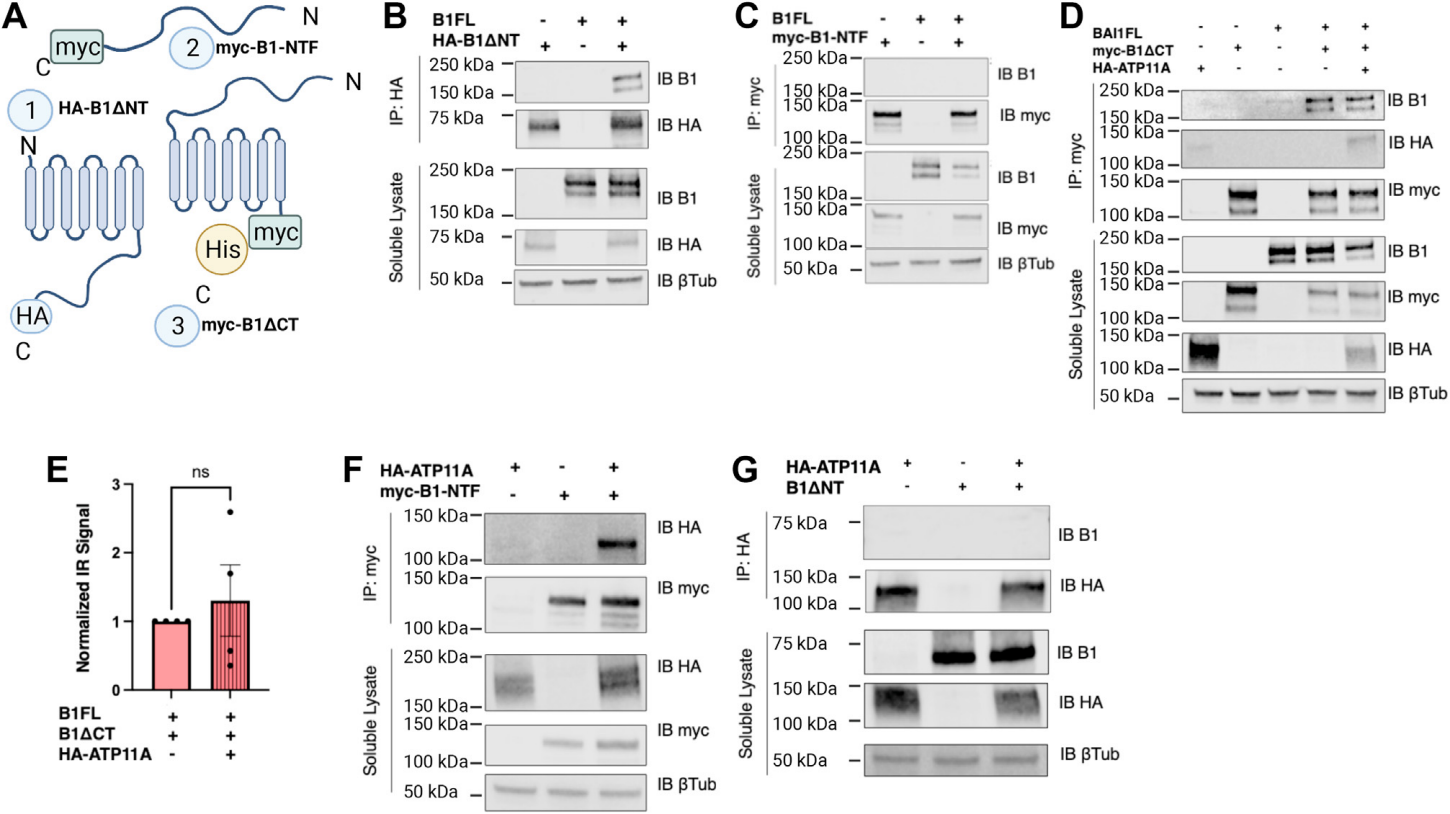
**B1 forms PS-independent multimers and also interacts with ATP11A.***A*, B1 schematics: B1 constructs used to evaluate multimer formation included (1) HA-tagged B1ΔNT, (2) myc-tagged B1-NTF, (3) His/myc-tagged B1ΔCT. *B*, Immunoprecipitation (IP) of HA-B1ΔNT resulted in co-IP of B1FL (n = 3). *C*, IP of myc-tagged B1-NTF did not result in any co-IP of B1FL (n = 3). “Soluble lysate” refers to the detergent-solubilized cell samples, which serve as transfection controls. *D*, IP of His/myc-B1ΔCT resulted in co-IP of B1FL in a manner that was not affected by coexpression with ATP11A (n = 4). Intriguingly, ATP11A itself also coimmunoprecipitated with His/myc-B1ΔCT. *E*, quantification of effect of ATP11A on B1FL-His/myc-B*1ΔC*T dimer formation: HA-ATP11A coexpression with B1FL and His/myc-B1ΔCT did not disrupt the ability of B1 to form multimers (normalized mean ± SEM is shown, unpaired *t* test, n = 4). *F*, IP of myc-tagged B1-NTF resulted in co-IP of HA-ATP11A (n = 3). *G*, IP of HA-ATP11A did not result in any detectable co-IP with B1ΔNT (n = 4). B1FL, full-length B1; HA, hemagglutinin; PS, phosphatidylserine.

PS has been proposed to modulate receptor function in some cases by promoting receptor clustering and multimerization (30, 31). Multimerization has been well documented for certain GPCRs (32, 33), but nothing is known about the potential multimerization of B1. To determine whether B1 forms multimers and whether this process might be influenced by PS, we leveraged several modified versions of B1 as shown in Figure 4*A*. When untagged B1FL and hemagglutinin (HA)-tagged B1ΔNT were coexpressed in HEK293T cells, we observed robust coimmunoprecipitation (co-IP) of these two receptor versions, suggesting that B1 multimerizes in a manner that does not require the NTF (Fig. 4*B*). In parallel experiments, B1FL was cotransfected with B1-NTF, but no co-IP was observed, consistent with the idea that the NTF of B1 does not participate in B1 multimer interaction (Fig. 4*C*). We also coexpressed B1FL with myc/His-tagged B1ΔCT, which lacks the majority of the B1 C terminus, and observed robust co-IP of these two receptors (Fig. 4*D*). It is important to note that the B1ΔCT construct lacks only the cytoplasmic C-terminal region of the receptor but retains the seven-TM region. Thus, taken together, these co-IP studies suggest that B1 forms multimers *via* its TM regions.

We next investigated whether B1 multimerization might be modulated by PS. Co-IP of B1FL and myc/His-tagged B1ΔCT was assessed in the absence and presence of ATP11A, which was shown earlier to reduce the levels of externalized PS. However, the presence of ATP11A was found to have no effect on B1 multimerization (Fig. 4*D*, with quantification in Fig. 4*E*). These findings suggested that B1 multimerization is independent of PS engagement by B1. Surprisingly, these experiments also yielded the observation that ATP11A itself robustly associates with myc/His-B1ΔCT (Fig. 4*D*).

### B1 interacts *via* its NTF region with ATP11A

To elucidate the structural determinants of the novel interaction that was serendipitously observed between B1 and ATP11A, we utilized the panel of truncated constructs described earlier and performed a series of co-IP assays. Given that both B1 and ATP11A contain numerous TM domains, and that B1 multimerization is dependent on these TM regions, we hypothesized that the B1–ATP11A interaction was most likely mediated *via* TM domain interactions. However, experiments assessing co-IP between B1-NTF and ATP11A unexpectedly revealed complex formation between ATP11A and B1-NTF, a truncated version of B1 that completely lacks the TM domains of the receptor (Fig. 4*F*). The B1-NTF is known to be secreted and remains associated with the outside of cells despite its lack of a TM domain (34), which helps to explain how this receptor fragment can possess the capacity to form stable complexes with a TM protein like ATP11A. Reciprocally, we coexpressed ATP11A with B1ΔNT, which *does* contain the TM domains of the receptor, and could not detect any co-IP of a B1ΔNT–ATP11A complex (Fig. 4*G*). This series of co-IP experiments indicated that the B1 association with ATP11A is mediated by the B1 NTF region.

### B1 signaling is reduced in cells lacking the scramblase ANO6

To further test the idea that B1 signaling activity is enhanced by receptor binding to externalized PS, we sought to assess B1 signaling under conditions where PS exposure was manipulated in a manner independent of ATP11A. As mentioned earlier, ANO6 is a lipid scramblase known to be endogenously expressed in HEK293T cells (23). Thus, we assessed B1 signaling in an *ANO6* KO cell line derived from HEK293T cells, which has been previously described (35, 36, 37, 38).

**Figure 5:**
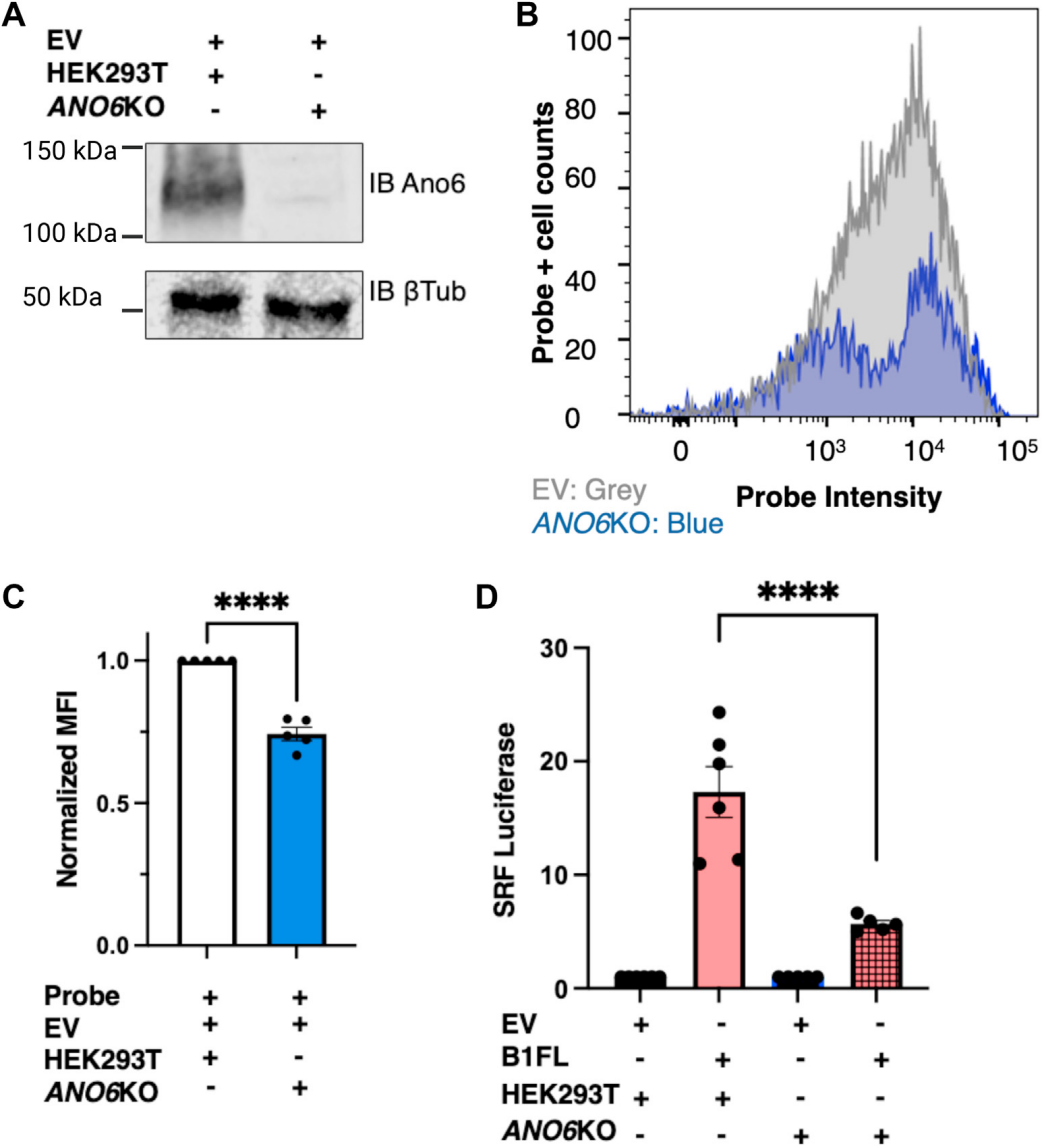
**B1 signaling activity is reduced in cells lacking ANO6.***A*, confirmation of *ANO6* KO in HEK293T cell line: on *left*, Western blot of wildtype HEK293T lysates *versus* lysates from *ANO6*KO cell line immunoblotted for ANO6 and β-tubulin. *B*, flow histogram of mock-transfected (*gray*) wildtype HEK293T cells (+annexin V) overlaid with mock-transfected *ANO6*KO cells (*blue*; +annexin V) demonstrates that *ANO6*KO cells exhibit lower baseline PS exposure than do wildtype HEK293T cells. Lower probe-positive cells were observed in the *ANO6*KO cells. *C*, quantification of PS exposure in *ANO6*KO mutant cell line compared with WT HEK293T cells: Mean fluorescence intensity (MFI) shown with *ANO6*KO condition normalized to mock-transfected condition. PS exposure was reduced by 26% in the *ANO6*KO cells (mean ± SEM is shown, unpaired *t* test, *p* = 0.0004, n = 5). *D*, B1FL activation of SRF-luciferase in *ANO6*KO cells was reduced by 67% relative to matched wildtype HEK293T cells (normalized to cell line, mean ± SEM is shown, ordinary one-way ANOVA with Tukey’s multiple comparisons test, *p* < 0.0001, n = 6 for HEK293T condition, n = 5 for *ANO6*KO condition, ROUT method used at 10% to remove one outlier from both conditions). ANO6, anoctamin 6; B1FL, full-length B1; HEK293T, human embryonic kidney 293T cell line; PS, phosphatidylserine.

We first confirmed *via* Western blot that these cells lack ANO6 expression (Fig. 5*A*) and also confirmed *via* flow cytometry that the *ANO6*KO cells exhibit significantly reduced PS exposure in comparison to wildtype HEK293T cells (Fig. 5*B*). Quantification of these flow data demonstrated that the *ANO6*KO cells exhibited a 26% reduction in PS exposure in comparison to wildtype HEK293T cells (Fig. 5*C*, unpaired *t* test with Welch’s correction, *p* = 0.0004).

With the *ANO6*KO cell line confirmed as having low basal PS exposure, B1FL G protein–dependent signaling to SRF-luciferase was measured (Fig. 5*D*). In comparison to B1FL signaling in HEK293T cells in matched experiments, B1FL G protein–dependent signaling in *ANO6*KO cells was lower by 67% (one-way ANOVA with Tukey’s multiple comparisons test, *p* < 0.0001). These findings provide further evidence, utilizing an independent method of altering PS exposure, that externalized PS promotes B1 signaling activity.

## Discussion

**Figure 6:**
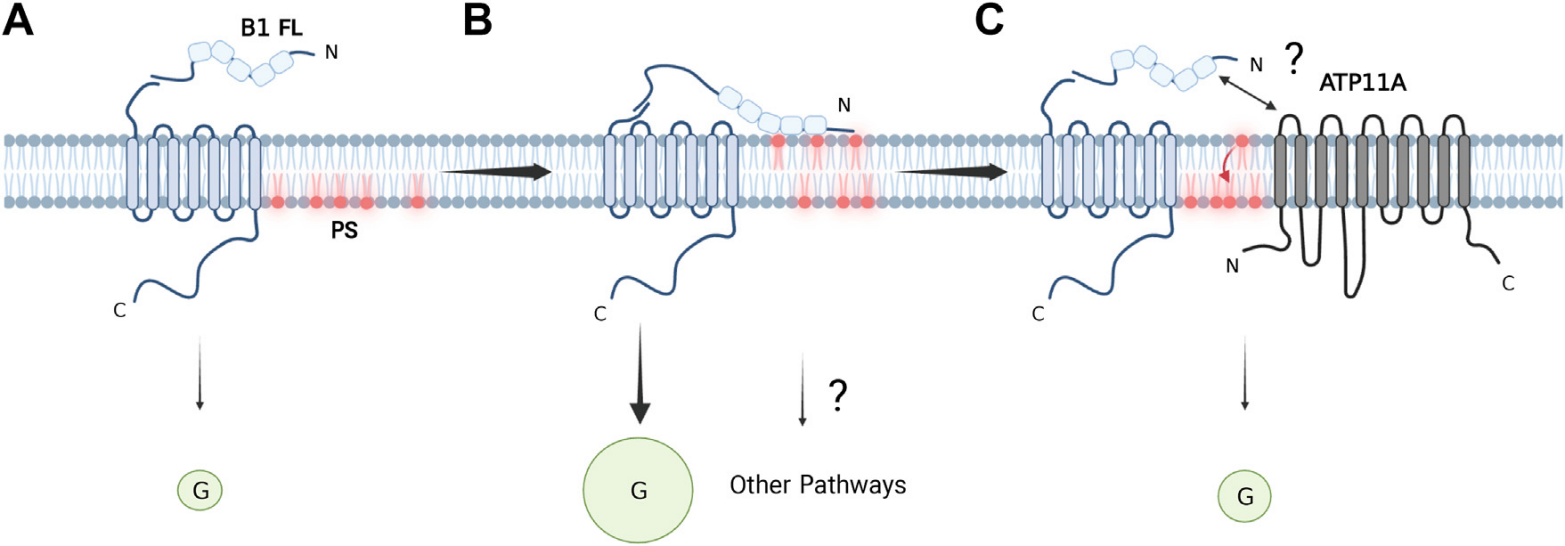
**Proposed mechanism for B1 interaction with PS and subsequent impact of ATP11A on B1 signaling.***A*, without PS engagement of the B1 NTF, B1 exhibits minimal G protein–dependent signaling. *B*, B1FL binds to externalized PS, which triggers a conformational change in the NTF and results in enhanced G protein–dependent signaling. *C*, the flippase ATP11A binds to B1 and also reduces PS exposure, thereby lowering B1 signaling activity when the flippase is active. NTF, N-terminal fragment; PS, phosphatidylserine.

B1 has long been known to bind PS in the context of macrophage-mediated engulfment of apoptotic cells (7), but the relationship of PS binding to B1 signaling activity has not been explored. The work described here demonstrates that B1 binding to externalized PS promotes the G protein–dependent signaling activity of the receptor. While modulation of PS externalization can exert pleiotropic effects on cells (19), our observation that altered PS exposure specifically affected signaling by B1FL, but not the truncated B1ΔNT that lacks the ability to bind PS, suggests that PS engagement by the B1 NTF was essential for the observed effects. B1 has previously been reported to exhibit high constitutive activity in HEK293T cells (13, 18), and the findings shown here reveal that at least a portion of this high constitutive activity is due to stimulation of B1 signaling by the basal level of exposed PS found in HEK293T cells (Fig. 6).

### Relevance of B1 engagement of PS to B1 function in the central nervous system

While HEK293T cells represent a useful model for studies on modulation of B1 signaling by PS, it is ultimately more physiologically important to understand whether (and how) PS might influence B1 signaling *in vivo*. PS exposure is increasingly appreciated as not only just a marker of programmed cell death but also an important cellular signal that can exert distinct physiological effects depending on how it is decoded by a diverse array of PS-binding receptors (20, 21, 22). For example, in the central nervous system (CNS), where B1 is most abundantly expressed, PS exposure is known to play a critical role in marking dendritic spines for pruning by phagocytic cells such as microglia and astrocytes (39, 40, 41, 42, 43).

If indeed B1 acts as a PS sensor in the CNS, then the function of the receptor *in vivo* may be fairly complex, given that B1 is known to be expressed in neurons (12, 13, 44, 45, 46), astrocytes (44, 45), and microglia (44, 47). Conceivably, the actions of B1 as a PS sensor in these distinct cell types may be very different or even opposing. For example, studies by Tolias *et al.* (14) have provided evidence (based on viral transduction of B1 into neurons *in vivo*) that neuronal B1 expression promotes the stability/maintenance of dendritic spines. Conversely, B1 expression in microglia has been shown to promote microglial engulfment of apoptotic cells (47) and may also plausibly mediate engulfment of dendritic spines (or entire synapses) that are marked for elimination because of externalization of PS (39, 40, 41, 42, 43). Similarly, astrocytes are also known to prune synaptic elements (48), and thus, astrocytic B1 may play a role in detecting externalized PS on postsynaptic spines and initiating pruning of those spines. Indeed, these opposing actions of B1 in distinct cell types may help to explain the mystery of why B1 knockdown in neurons decreases the number of dendritic spines (12, 14), yet mice lacking B1 globally in all cell types exhibit no discernible changes in spine density (16). Conceivably, B1 in neurons may detect PS as a stress signal and act to protect dendritic spines, whereas B1 found in glia (microglia and astrocytes) may detect PS as an engulfment signal and thereby facilitate spine pruning. These ideas may be tested in future studies in which B1 is deleted *in vivo* in a cell-specific manner.

### PS binding by other AGPCRs

While B1 was the first AGPCR identified as a PS-binding receptor, another AGPCR, GPR56 (ADGRG1), was also recently shown by Piao *et al.* (42) to bind PS in a manner that facilitates synaptic refinement by microglia. Interestingly, there are several notable differences between B1 and GPR56 as PS sensors. First, B1 binds PS *via* its TSRs (7), whereas GPR56 binds PS *via* its GAIN domain (42). Most AGPCRs possess GAIN domains (2, 3, 4), but at this point, it is unknown whether PS binding is a common property of GAIN domains or whether this property is unique to the GAIN domain of GPR56. Future structural studies to elucidate the structural determinants of PS binding by B1 and GPR56 may facilitate predictions about potential PS binding by other AGPCRs. Second, B1 and GPR56 exhibit distinct cellular distributions within the CNS: as described previously, B1 is expressed in neurons, astrocytes, and microglia, whereas GPR56 is not expressed at all in mature neurons but rather is expressed in oligodendrocytes, microglia, astrocytes, and neuronal precursor cells (44, 49, 50).

Third, the aforementioned findings in our *Results* section demonstrate that B1 engagement of externalized PS stimulates the signaling activity of the receptor, whereas the effect of PS on GPR56 signaling has not yet been explored. Thus, it will be interesting in future studies to dissect the differential roles in PS detection played by B1 *versus* GPR56 and also assess whether other AGPCRs have the ability to act as PS sensors.

### Physiological relevance of ATP11 flippases in the CNS

The studies described here utilized the PS flippase ATP11A as a tool to manipulate PS exposure in HEK293T cells. Intriguingly, far from just being a tool that has no relation to brain physiology, ATP11A and related flippases are well known to be localized to dendritic spines *in vivo* and to regulate synaptic plasticity. For example, KO of the PS flippase ATP11B, a close relative of ATP11A, results in striking perturbations to dendritic spine morphology and hippocampal synaptic plasticity (51). Similarly, knockdown of CDC50A, a chaperone protein required for proper trafficking of ATP11 family of phospholipid flippases (26), was shown to result in increased PS exposure at synapses and aberrant pruning by microglia (52). Moreover, the synaptic function of the ATP11 family of flippases is clinically relevant, given that a *de novo* heterozygous ATP11A point mutation was recently found to result in severe developmental delays and neurological deterioration (53).

The well-established synaptic actions of the ATP11 family of flippases, in conjunction with our data shown here that ATP11A modulates B1 activity and that ATP11A and B1 can robustly associate, suggest the possibility that B1 and ATP11 flippases form complexes at synapses that may act synergistically to sense and regulate PS levels in dendritic spines (Fig. 6*C*).

### B1 binds multiple ligands

Although B1 may serve as a PS sensor at synapses and in other cellular contexts, this by no means suggests that PS is the sole ligand for B1. Most AGPCRs possess massive extracellular NTF regions featuring numerous modular domains, and it seems highly likely that these multiple domains engage with multiple ligands (2, 3, 4). In the case of B1, for example, it has been shown that the B1 NTF binds with high affinity to reticulon-4 receptors to regulate dendritic arborization and synapse formation (34, 54). B1 also has been reported to bind to a number of other extracellular and/or TM proteins, including integrins (55), CD36 (56), the complement-like protein C1q (57), and neuroligin-1 (14). In future studies, it will be interesting to examine the potential interplay between B1 engagement of PS *versus* B1 engagement of reticulon-4 receptors and other B1 ligands, including ligands that may yet be undiscovered.

### The pharmacology of AGPCRs

AGPCR signaling is controlled by dynamic changes in the association between the receptors’ NTF and CTF regions, with mechanical forces on the NTF changing the position of the stalk region (also known as the “tethered agonist” or “stachel”) to modulate receptor signaling activity (2, 3, 4). Some AGPCRs are also activated by small-molecule ligands such as steroids (58, 59) or bioactive lipids (60), leading to the emerging view in the field that AGPCRs serve as massive signaling platforms that are crucial for the integration of adhesive, mechanosensory, and chemical stimuli (3).

When AGPCRs are found to bind multiple ligands, as in the case of B1, it naturally raises questions about whether one of the ligands might be the true orthosteric agonist, with other ligands providing allosteric modulation of signaling. However, in comparison to traditional ligand–receptor complexes, wherein it is quite clear where and how ligand binding to a GPCR can trigger signal transduction, AGPCR activation at this point is more nebulous because of the ability of the large NTF regions to bind so many different molecules. Given the complexity of AGPCR signaling and the emerging view that these receptors are massive platforms that integrate a variety of signals, it may be that traditional pharmacological terms like “orthosteric agonist” or “allosteric modulator” are simply not appropriate to describe the multiligand binding nature of this receptor class. This classical terminology may not fully capture the complexity of AGPCR signaling, in that one NTF region can bind to multiple ligands to trigger different receptor responses in different cell types. Thus, the pharmacology of AGPCR signaling remains murky at present, and determining a single “orthosteric agonist” for the members of this receptor class may not be possible. Perhaps instead, it would be preferable to refer to modulators of AGPCR signaling simply as “ligands” at this point and not try to become more specific until the interplay of the various ligands can be evaluated and high-resolution structures can shed more light on the active conformations that can be achieved by the members of this receptor class.

### Future studies

The work presented here demonstrates that B1 binds PS, and that this lipid engagement alters the receptor’s signaling. In future studies, it would be of interest to understand whether B1 itself regulates PS externalization, such that B1 might serve as a PS sensor that provides real-time feedback to regulate exposure of PS. In addition, as described previously, it would be of great interest to study the importance of B1 recognition of PS in different cellular contexts (*i.e.*, neurons *versus* astrocytes *versus* microglia in the CNS). It would also be interesting in future work to measure whether PS engagement by B1 alters the receptor’s ability to engage in key protein–protein interactions with cytoplasmic binding partners other than G proteins. In addition to its G protein coupling, B1 is also known to bind intracellularly to beta-arrestins (13, 18), IRSp53 (61), MDM2 (16, 62), MAGI-3 (13), Tiam-1 (12, 14, 15, 63), Bcr (15), and PSD-95 (13, 16). What is the effect of B1 PS engagement on B1 interactions with these various cytoplasmic binding partners? Does PS binding by B1 lead only to enhanced G protein–dependent signaling, such that PS serves as a “biased ligand” (64, 65, 66), or does PS equally promote all signaling pathways downstream of B1? Future studies along these lines would help to expand our knowledge of AGPCR signaling in general and in particular enhance our understanding of the ability of B1 to serve as a PS sensor.

## Experimental procedures

### Constructs

Human B1FL (1–1584), B1 ΔNT (927–1584) (and HA-B1 ΔNT), B1 myc-ΔCT (1–1200), and B1-NTF (1–927; also known as “Vstat120”) constructs have been described previously (13, 18, 62). The latter two constructs were kindly provided by Erwin Van Meir (University of Alabama at Birmingham). Human HA-ATP11A and HA-ATP11A (E186Q) have also been described previously (26, 27, 29, 67, 68, 69, 70, 71, 72, 73, 74).

## Cell culture

HEK293T cells were acquired from American Type Culture Collection and maintained in Dulbecco's modified Eagle's medium (Thermo Fisher) supplemented with 10% fetal bovine serum (Rockland) and 1% penicillin/streptomycin (VWR) in a humid, 5% CO_2_, 37 °C incubator. Cells were transfected with polyethyleneimine (PEI) or Mirus TransIT-LT1 (Mirus Bio) according to the manufacturer’s protocol. The *ANO6*KO cell line was kindly provided by Huanghe Yang (Duke University) and developed as described (35, 36, 37, 38).

### Luciferase reporter assay

HEK293T cells were seeded into 96-well plates (Corning) at 50,000 cells per well 20 to 24 h prior to transfection. Each well was transfected with 50 ng SRF-luciferase (a reporter of RhoA signaling *via* G⍺12/13, pGL4.34; Promega), 1 ng Renilla luciferase, and 50 ng receptor or empty vector (EV) DNA, as previously described (17).

At 48 h after transfection, Dual-Glo luciferase assay was performed according to the manufacturer’s protocol by adding luciferase reagent (Promega) to cells for 10 min in the dark at room temperature and read on FLUOstar Omega (BMG Labtech). Next stop-and-glo reagent (Promega) was added to stop the reaction for the Renilla luciferase read after another 10 min incubation in the dark and at room temperature (also read on the same plate reader). Results were calculated for each assay by determining the luminescence ratio of firefly:Renilla luciferase counts, normalized to EV-transfected wells.

### Coculture experiments

HEK293T cells were seeded into 96-well plates at 50,000 cells per well 20 to 24 h prior to transfection using Mirus or PEI. Concurrently, a 10 cm dish was also plated and transfected with EV DNA. In the 96-well dish, each well was then transfected with reporter (SRF-luciferase), Renilla, and receptor or EV DNA. At 24 h after transfection, 50,000 cells were collected and counted from the 10 cm dish and plated onto half of the wells in the 96-well plate to observe whether the additional cells altered the signaling of the B1FL-positive cells. Signaling was then measured 48 h after transfection.

### Western blot

Protein samples were reduced and denatured in Laemmli buffer, loaded into 4 to 20%, Tris–glycine gels (Bio-Rad) for SDS-PAGE, and then transferred to nitrocellulose membranes (Bio-Rad). Blots were blocked with EveryBlot blocking buffer (Bio-Rad) and incubated while shaking with primary antibodies (specific antibodies listed later, all used at 1:1000 dilution) overnight at 4 ^°^C. Goat anti-rabbit immunoglobulin G or goat antimouse immunoglobulin G secondary antibody (IRDye 800CW, 1:5000 dilution; Licor) was then used to amplify signal (1 h incubation, shaking at room temperature), and blots were imaged on a Licor Fc machine. ImageStudio (Licor) was used for quantification of bands on the resultant Western blots. The primary antibodies used were anti-BAI1 (Thermo Fisher; catalog no.: PA1-46465, host: rabbit), anti-HA (Cell Signaling Technology; catalog no.: C29F4, host: rabbit), anti-Myc (Cell Signaling Technology; catalog no.: 9B11, host: mouse), anti-β-tubulin (Cell Signaling Technology; catalog no.: 2146S, host: rabbit), and anti-ANO6 (Invitrogen; catalog no.: PA5-58610, host: rabbit).

### Co-IP

At 48 h after transfection, 10 cm plates containing HEK293T cells were washed with cold PBS + 0.9 mM Ca^2+^ and solubilized in 1 ml harvest buffer (150 mM NaCl, 25 mM Hepes, pH 7.2, 1 mM EDTA, 1% Triton X-100, and 1× HALT protease/phosphatase inhibitors) overnight at 4 °C, end over end. Next, unsolubilized material was cleared by centrifugation (15 min at 13,500 rpm, 4 °C), and 90 μl supernatant was collected for blotting (mixed 1:1 with 2× Laemmli buffer; Bio-Rad), whereas the remainder (∼910 μl) was mixed with washed beads (either anti-HA or anti-Myc agarose beads from Pierce). The lysate–bead mixture was rotated end over end overnight at 4 °C. Next, beads were briefly centrifuged in a table-top centrifuge, washed 3× in harvest buffer, and eluted in Laemmli loading buffer before loading on 4 to 20% Tris–glycine gels for SDS-PAGE and Western blotting. Western blot bands were quantified using ImageStudio software.

### Cell surface biotinylation

The Pierce Cell Surface Biotinylation and Isolation kit (Thermo; catalog no.: A44390) was used according to the manufacturer’s protocol to evaluate receptor presence in the plasma membrane. Briefly, 48 h after transfection, 10 cm plates of transfected HEK293T cells were placed on ice and washed with ice-cold PBS before being incubated with membrane-impermeant Sulfo-NHS-SS-Biotin in PBS for 10 min to biotinylate the surface. Cells were then washed in TBS three times, lysed in kit-provided buffer, and cleared by centrifugation. The lysates were incubated with NeutrAvidin Agarose beads. Beads were then washed three times in the manufacturer’s wash buffer and resuspended in Laemmli buffer. Biotinylated proteins were detected *via* Western blotting.

### Flow cytometry

The Cell Meter Phosphatidylserine Apoptosis Assay Kit (Green Fluorescence Optimized for Flow Cytometry; from AAT Bioquest; catalog no.: 22824), which utilizes annexin V to detect externalized PS, was used according to the manufacturer’s protocol. Briefly, HEK293T cells or *ANO6*KO cells were plated in 10 cm dishes and transfected using PEI with 4 μg EV, ATP11A, or ATP11A (E186Q). About 48 h later, cells were collected into Dulbecco's modified Eagle's medium containing 10% fetal bovine serum and 1% penicillin/streptomycin, triturated, and counted. Note: no trypsin was used when collecting the transfected cells in this step to avoid protease-mediated cleavage of B1. Instead, mechanical dissociation was used in complete media to obtain single-cell suspension of cultured cells. Next, 5 × 10^5^ cells were aliquoted per experimental condition, spun down, and resuspended in kit-provided proprietary assay buffer with annexin V probe and incubated for a minimum of 30 min in the dark before analysis by flow cytometry on a FACSymphony A3 5-Laser Cell Analyzer. Single-cell populations of cells were identified using side scatter width and side scatter height. To gate for saturation of signal, A23187 was used to induce PS exposure and served as positive control in parallel with same gates used in all experiments run (MilliporeSigma). Negative control was gated for lack of signal using cells without probe. Once positive and negative control were used to establish voltage and gating, these same settings were used for all experiments described. A total of 20,000 events were recorded for each sample.

### Quantification and data analysis

GraphPad Prism (GraphPad Software, Inc) was used to analyze data. Ordinary one-way ANOVA with Tukey’s multiple comparisons test or unpaired *t* tests were used to determine statistically significant differences among experimental conditions. Where normalized data were used, Welch’s correction was implemented in addition to the unpaired *t* test. ROUT method was used at 10% to identify any outliers in signaling assays (and indicated in legend where outliers were appropriately removed). Sample sizes are reported in the *Results* section; n values refer to the number of biological replicates for each set of experiments.

## Data availability

The datasets generated during the current study are available from the corresponding author upon reasonable request.

## Conflict of interest

The authors declare that they have no conflicts of interest with the contents of this article.
